# Characterization of Age-Dependent and Progressive Cortical Neuronal Degeneration in *Presenilin* Conditional Mutant Mice

**DOI:** 10.1371/journal.pone.0010195

**Published:** 2010-04-15

**Authors:** Mary Wines-Samuelson, Eva C. Schulte, Miriam J. Smith, Chiye Aoki, Xinran Liu, Raymond J. Kelleher, Jie Shen

**Affiliations:** 1 Center for Neurologic Diseases, Department of Neurology, Brigham and Women's Hospital, Harvard Medical School, Boston, Massachusetts, United States of America; 2 Center for Neural Science, New York University, New York, New York, United States of America; 3 Department of Neuroscience, University of Texas Southwestern Medical Center, Dallas, Texas, United States of America; 4 Center for Human Genetic Research, Massachusetts General Hospital, Harvard Medical School, Boston, Massachusetts, United States of America; National Institutes of Health, United States of America

## Abstract

Presenilins are the major causative genes of familial Alzheimer's disease (AD). Our previous study has demonstrated essential roles of presenilins in memory and neuronal survival. Here, we explore further how loss of presenilins results in age-related, progressive neurodegeneration in the adult cerebral cortex, where the pathogenesis of AD occurs. To circumvent the requirement of presenilins for embryonic development, we used *presenilin* conditional double knockout (*Psen* cDKO) mice, in which presenilin inactivation is restricted temporally and spatially to excitatory neurons of the postnatal forebrain beginning at 4 weeks of age. Increases in the number of degenerating (Fluoro-Jade B+, 7.6-fold) and apoptotic (TUNEL+, 7.4-fold) neurons, which represent ∼0.1% of all cortical neurons, were first detected at 2 months of age when there is still no significant loss of cortical neurons and volume in *Psen* cDKO mice. By 4 months of age, significant loss of cortical neurons (∼9%) and gliosis was found in *Psen* cDKO mice. The apoptotic cell death is associated with caspase activation, as shown by increased numbers of cells immunoreactive for active caspases 9 and 3 in the *Psen* cDKO cortex. The vulnerability of cortical neurons to loss of presenilins is region-specific with cortical neurons in the lateral cortex most susceptible. Compared to the neocortex, the increase in apoptotic cell death and the extent of neurodegeneration are less dramatic in the *Psen* cDKO hippocampus, possibly in part due to increased neurogenesis in the aging dentate gyrus. Neurodegeneration is also accompanied with mitochondrial defects, as indicated by reduced mitochondrial density and altered mitochondrial size distribution in aging *Psen* cortical neurons. Together, our findings show that loss of presenilins in cortical neurons causes apoptotic cell death occurring in a very small percentage of neurons, which accumulates over time and leads to substantial loss of cortical neurons in the aging brain. The low occurrence and significant delay of apoptosis among cortical neurons lacking presenilins suggest that loss of presenilins may induce apoptotic neuronal death through disruption of cellular homeostasis rather than direct activation of apoptosis pathways.

## Introduction

*Presenilins* (*Psen 1 and 2*) are the major causative genes of early-onset familial Alzheimer's disease (FAD) and harbor ∼90% of the identified FAD-linked mutations. Presenilins are broadly expressed and play essential roles during embryonic development [1], [2], [3], [4]. Specifically, presenilins are required for maintenance of neural progenitor population as well as neurogenesis and neuronal migration [4], [5], [6], [7], [8]. To circumvent the requirement of presenilins in development, we previously generated a *presenilin* conditional double knockout (*Psen* cDKO) mouse, in which presenilin inactivation is restricted spatially and temporally to excitatory neurons of the postnatal forebrain using the Cre/loxP technology [9], [10]. Thus, *Psen* cDKO mice permit assessment of direct consequences of presenilin inactivation in excitatory pyramidal neurons of the adult cerebral cortex, where presenilins are normally expressed highly and AD pathogenesis occurs. Analysis of these mutant mice demonstrated that loss of presenilins in mature neurons of the cerebral cortex results in progressive impairment in synaptic plasticity and learning and memory, followed by age-dependent neurodegeneration [10], [11].

In *Psen* cDKO mice at 2 months of age, approximately one month after presenilin inactivation, memory impairment as well as specific presynaptic and postsynaptic defects were found in the absence of significant loss of cortical neurons or volumes [10]. By 6 and 9 months of age, 18% and 24% of cortical neurons were lost, respectively [10]. These results were further supported by an independent study using a similar Cre line, which found elevated levels of gliosis and decreased cortical volume at 10 months of age [12]. Presenilins promote memory and neuronal survival in a γ-secretase-dependent manner, as conditional inactivation of another component of the γ-secretase complex, nicastrin, results in similar patterns of memory impairment and age-related neurodegeneration [13]. However, the precise time of the onset of neuronal degeneration and the mode of neuronal death were less clear in *Psen* cDKO mice.

In the current study, we show how loss of presenilin function in the adult brain leads to age-dependent neurodegeneration. Cell death induced by loss of presenilins begins at 2 months of age in the cerebral cortex via apoptosis. Remarkably, only a very small percentage of cortical neurons undergo apoptotic cell death at any given time point, though over time an increasingly higher percentage of cortical neurons are lost. Interestingly, hippocampal neurons are less vulnerable to cell death induced by loss of presenilins, whereas lateral cortical neurons are particularly vulnerable, suggesting brain subregion specificity for presenilin-dependent neuronal survival.

## Materials and Methods

### Mice

Generation of *Psen* cDKO (*fPS1/fPS1;PS2−/−;Cre*) mice was described previously [10]. *fPS1/fPS1;PS2−/−;Cre* mice were bred with *fPS1/fPS1;PS2−/−* mice to obtain more cDKO mice (*fPS1/fPS1;PS2−/−;Cre*) and *fPS1/fPS1;Cre* were bred with *fPS1/fPS1* to obtain control mice (*fPS1/fPS1*). The genetic background of these mice was similar in the C57BL6/129 hybrid background with breeding carried out similarly for both groups.

### Tissue preparation

To prepare brain samples for immunostaining, Nissl and Fluoro-Jade B staining, and TUNEL labeling, mice were euthanized with carbon dioxide and perfused with 4% paraformaldehyde in phosphate-buffered saline (PBS). After perfusion, brains were removed and postfixed 2 hours at 4°C in the same fixative. Each brain was bisected sagittally, such that one hemisphere was prepared for paraffin embedding while the other was cryoprotected by immersion in 30% sucrose/PBS overnight at 4°C and embedded in OCT for frozen sectioning. For Nissl staining following by stereological neuron counting, 10 µm thick paraffin sections were collected; for immunostaining, Fluoro-Jade B, and TUNEL analysis, 20 µm thick sections were collected on a cryostat.

### Neuron count

10 µm thick parasagittal paraffin sections were stained with cresyl violet, and neuron number was estimated using the fractionator and optical dissector methods under an Olympus BX51 light microscope equipped with a CCD camera connected to a computer running Bioquant image analysis software. Cortical volume was also estimated using the same software and equipment. The experimenter was blind to the genotypes of the mice. Statistical significance was determined using Student's *t* test.

### Immunostaining and quantification

20 µm thick frozen sections were rinsed in PBS followed by antigen retrieval in boiling citrate buffer. Sections were blocked with 5% normal goat serum (NGS), 0.03% TritonX-100 in PBS for 1 hour at room temperature, and incubated in primary antibody diluted in blocking buffer overnight at 4°C. The following antibodies were used: rabbit anti-cleaved caspase-9 (Asp353) (Cell Signaling Technology, 1∶200), and rabbit anti-cleaved caspase-3 (Asp175) (Cell Signaling Technology, 1∶100). Primary antibodies were detected with fluorescent secondary antibodies (Alexa Fluor 488 or 594, Molecular Probes) diluted 1∶300 in 5% NGS/PBS. Images of stained sections were captured on a Zeiss LSM510 laser-scanning confocal microscope. Quantification was performed blind to genotype on a series of sections from medial to lateral in each brain, and then the average number of positive cells per section was determined for each.

### TUNEL labeling and quantification

For labeling of brain sections, a series of 10 frozen sagittal sections at approximately 300 µm intervals were stained and quantified per brain. TUNEL labeling was performed using the Roche *In Situ* Cell Death Detection Kit as per the manufacturer's recommended protocol, with the following modifications: antigen retrieval was performed using boiling citrate buffer; TdT enzyme was diluted 1∶20 in labeling mix; and slides were blocked for 30 min at room temperature prior to TUNEL labeling with buffer containing 10% normal goat serum, 3% BSA in 0.1 M Tris pH 7.5. All positive cells were counted per section in the neocortex and hippocampus by an experimenter blind to genotype. The number of positive cells per 20 µm-thick section was determined, and then averaged per genotype.

### Electron microscopy and quantitative analysis

Three pairs of age-matched mice (3 control, 3 *PS* cDKO, 5 months old) were perfused with 1% glutaraldehyde and 2% paraformaldehyde in 0.1 M phosphate buffer. Brains were removed and immersion fixed in 2.5% glutaraldehyde and 2% paraformaldehyde in 0.1 M cacodylate buffer overnight at 4°C. The 150 µm-thick sections were made by a vibratome and post-fixed in 1% OsO_4_, 0.8% potassium ferricyanide in the same buffer for one hour at room temperature. Specimens were stained en bloc with 2% aqueous uranyl acetate for 15 min, dehydrated in a graded series of ethanol to 100% and embedded in Poly/bed 812 (Polysciences Inc., Warrington, PA). Thin sections (60 nm) were made by a Leica Ultracut microtome and post-stained with uranyl acetate and lead citrate. The sample grids were examined with an FEI Tecnai transmission electron microscope at 120 kV of accelerating voltage, and the digital images were captured with a SIS Morada CCD camera.

For quantification of mitochondria, 13,000× magnification images were collected and imported into ImageJ. The scale of magnification/pixel density was calibrated, and each mitochondrion per image was counted and its area quantified (ImageJ). The total number of mitochondria per image per genotype was scored, and the average number per genotype was calculated and compared. The size distribution of mitochondria per image was determined by assignment to arbitrary bins based on area. Three pairs of mice age 5–6 months were analyzed.

## Results

### Time course of neurodegeneration in the cerebral cortex of *Psen* cDKO mice

**Figure 1:**
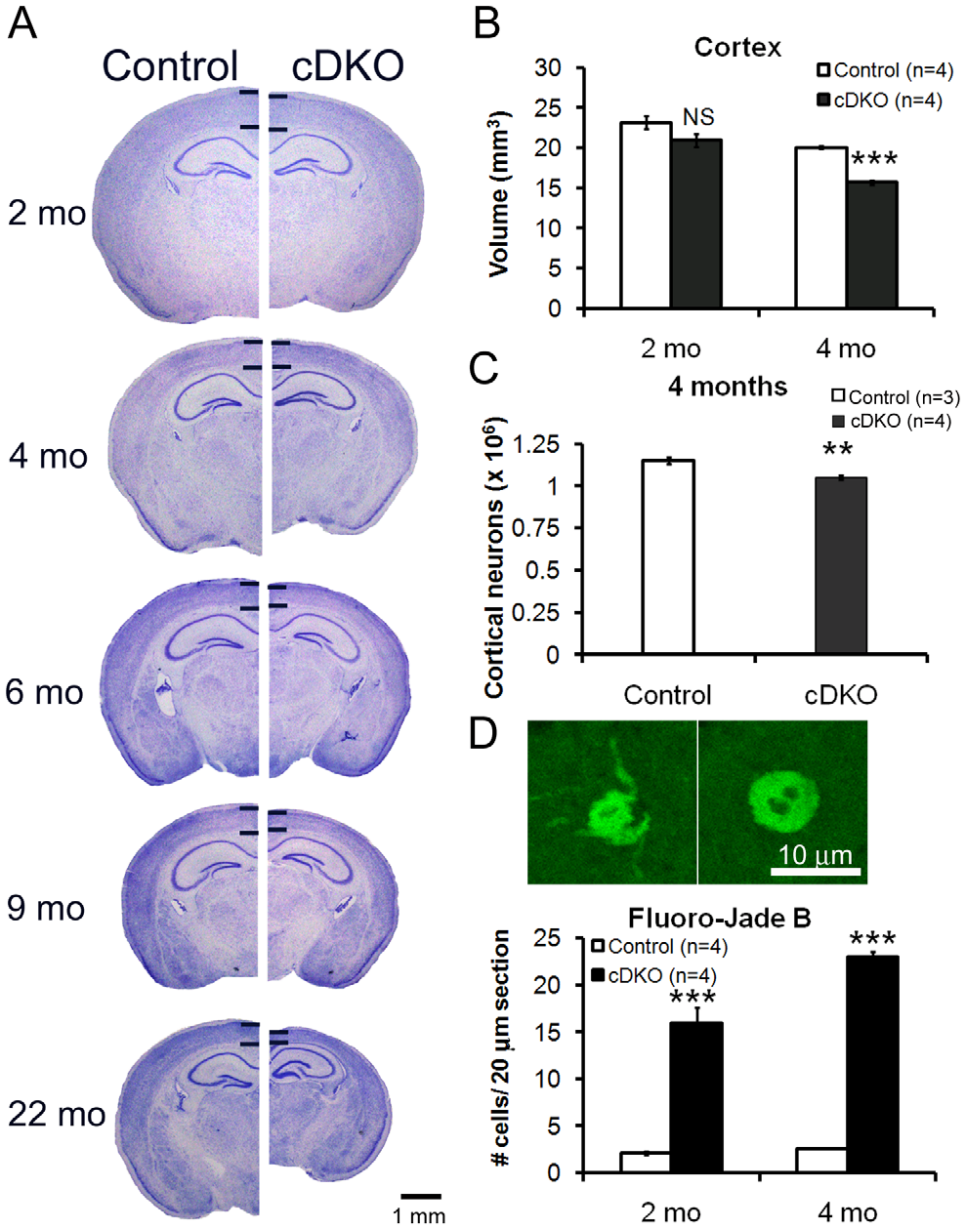
Increases in degenerating cortical neurons at 2 months followed by significant loss of cortical neurons and volume at 4 months in *Psen* cDKO mice. (*A*) Nissl-stained images of coronal sections from age-matched control (*left*) and *Psen* cDKO mutant (*right*) brains from 2 to 22 months of age are shown. Black horizontal bars delineate neocortical layers. At 2 months, no detectable difference is found in size or shape of the *Psen* cDKO brain relative to control. However, subsequent ages reveal a gradual decrease in cortical thickness in *Psen* cDKO mice. Scale bar: 1 mm. (*B*) Stereological measurement of cortical volume from control (open bars) and *Psen* cDKO mutant (black bars) brains at 2 and 4 months. Values are presented per hemisphere. At 2 months of age, the cortical volume is similar between control and cDKO mice (p>0.05). At 4 months, the cortical volume in cDKO mice is significantly smaller (21.7% reduction; p<0.001). NS, not significant; ***, p<0.001; n = 4 mice per genotype. (*C*) Stereological quantification of neuron number in the neocortex. At 4 months, an 8.7% decrease in neuron number (1.15×10^6^) is present in *Psen* cDKO mice, relative to controls (1.05×10^6^; **p<0.01)(n = 3 control mice +4 cDKO mice). (*D*) Quantification of degenerative neurons. *Top*: Fluoro-Jade B labels degenerating neurons in brain sections, and high magnification (100×) confocal images of single cells stained with Fluoro-Jade B. Scale bar: 10 µm. *Bottom*: Increased numbers of Fluoro-Jade-positive neurons are detected at 2 months in the *Psen* cDKO cortex (7.6-fold increase; p<0.0001) relative to control, with even greater numbers of dying neurons present by 4 months of age (9.0-fold increase; p<0.0001)(n = 4 mice per genotype per age; 10 sections analyzed per mouse). Data are presented as the mean ± s.e.m.

To evaluate age-related neurodegeneration caused by loss of presenilins, we performed histological analysis of postnatal forebrain-specific *Psen* cDKO (*fPsen1/fPsen1; Psen2−/−; αCaMKII-Cre*) and control (*fPsen1/fPsen1*) mice from 2 to 22 months of age (Fig. 1A). We previously showed that expression of *Psen1* mRNAs and proteins are unaltered in *fPsen1/fPsen1* mice relative to wild-type mice, and that presenilin-1 inactivation begins at 3–4 weeks of age postnatally [14]. Comparison of comparable Nissl stained brain sections revealed a subtle thinning of the cerebral cortex at 4 and 6 months of age, and a striking reduction in the size of the cortex by 22 months of age (Fig. 1A). Quantitative stereological analysis showed similar cortical volume in *Psen* cDKO mice at 2 months and a significant reduction at 4 months (21.7%, p<0.0001) (Fig. 1B; Table 1). Thus, inactivation of presenilins causes age-related, progressive loss of cortical volume with no significant loss at 2 months, ∼22% loss at 4 months, ∼35% loss at 6–9 months and ∼53% loss at 22 months (Table 1).

**Table 1:** Quantitative analysis of neurodegeneration and cell death in *Psen* cDKO Cx† ‡.

Parameter	2 mo	4 mo	6 mo	9 mo	22 mo
Volume	NS#	−21.7%	−35%	−35%*	−52.7%
Neuron number	NS*	−8.7%	−18%*	−24%*	ND
Fluoro-Jade B+	0.08%	0.55%	ND	ND	ND
TUNEL+	0.13%	0.58%	ND	ND	ND

† Values for Fluoro-Jade B and TUNEL represent percentage of positive cells based on stereological estimates of total cell number. Note that although *Psen1* gene inactivation, measured by *Psen1* mRNA levels, occurs by 3–4 weeks, death is not increased at this timepoint. Since Psen1 protein levels persist and gradually diminish between P22 and 6 weeks [14], the delay in onset of death is likely due to the continued presence of Psen1 protein subsequent to gene inactivation.

‡ All calculations of total % death are based on stereological estimates of cortical cell number at 2 months of age (4.8×10^6^ cells/hemisphere; [10] and at 4 months of age (Fig. 1) combined with an estimate of 5 mm width of one hemisphere. Raw data (# dead cells/20 µm-thick sagittal section) was converted to a % by the following calculations: 1) the fraction of a hemisphere represented by one 20-µm thick section (1 section =  20 µm/5000 µm, or 1/250^th^ of a hemisphere), 2) the average number of cortical cells per 20 µm -thick section (4,800,000 cells/hemisphere (2 mo) or 1,500,000 cells/hemisphere (4 mo) ×1/250^th^ of a hemisphere  = 19,200 cells/20 µm section), and 3) the percentage of dying cells per section (average number of dead cells per 20 µm section/19,200 total cells).

#actual value = −9.6% (p = 0.09; n = 4 pairs); *ND*, not determined. *[10]

We further quantified the number of neurons in the cerebral cortex of *Psen* cDKO mice at 4 months of age, as we previously found no significant reduction in the number of cortical neurons at 2 months, and loss of 18% and 24% of cortical neurons at 6 and 9 months, respectively [10]. Neuron count using stereological methods identified a small but significant decrease in cortical cell number in *Psen* cDKO at 4 months (8.7%, p<0.01; Fig. 1C; Table 1). These data indicate progressive neuronal cell loss in the absence of presenilins in the cerebral cortex.

To assess neurodegeneration further in *Psen* cDKO mice, we used Fluoro-Jade B staining, which detects degenerating neurons [15], [16], [17], [18], to quantify the number of degenerating neurons in *Psen* cDKO mice at 2 and 4 months of age (Fig. 1D; Table 1). In the neocortex of *Psen* cDKO mice, the number of degenerating neurons was significantly increased at 2 months (7.6-fold; p<0.00001) and 4 months of age (9.0-fold; p<0.00001), compared to the control (Fig. 1D). Thus, while the total number of cortical neurons and the size of the cortical volume are not significantly altered at 2 months of age in *Psen* cDKO mice, the number of degenerating neurons is dramatically increased. However, despite the large increases in degenerating neurons in *Psen* cDKO mice, the absolute number of degenerating neurons is still rather small (∼15–20 cells in the 20 µm sagittal section in the neocortex), representing ∼0.1% of neurons at 2 months of age (Table 1).

### Apoptotic cell death in the cerebral cortex of *Psen* cDKO mice

**Figure 2:**
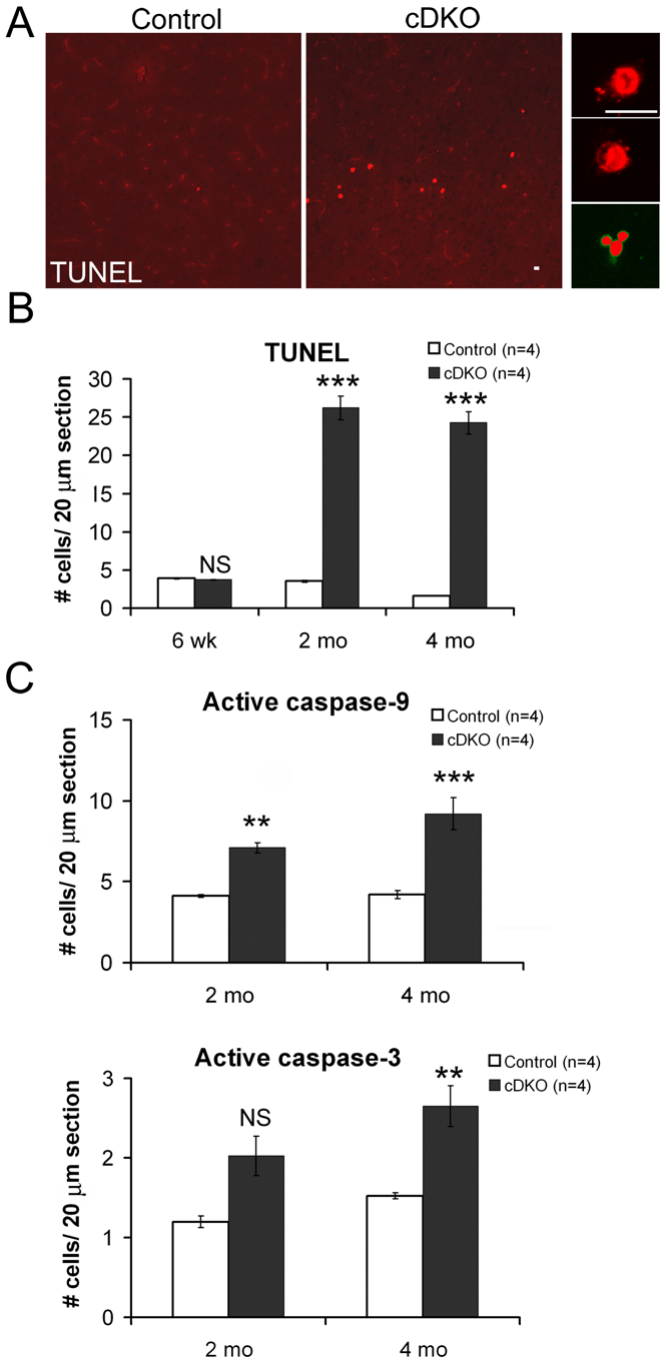
Apoptotic neuronal death in the cerebral cortex of *Psen* cDKO mice. (*A*) Left: Confocal images of TUNEL-stained cells in the neocortex of control and cDKO mice at low magnification (20×). Right: High magnification (100×) images of individual TUNEL+ cells. Scale bar: 10 µm. (*B*) Quantification of TUNEL+ cells at the ages of 6 weeks, 2 and 4 months. At 6 weeks, similar low numbers of TUNEL+ cells are present in the neocortex between control and *Psen* cDKO mice. In contrast, dramatic increases in TUNEL+ cells are observed in the cDKO neocortex at 2 months (7.4-fold increase) and 4 months (15.2-fold increase)(n = 4 mice per genotype per age; 10 sections analyzed per mouse). (*C*) Quantification of cells positive for activated caspase-9 or caspase-3. The number of active caspase-9+ cells is significantly increased in the neocortex of cDKO mice at 2 and 4 months of age (2m: p<0.005; 4m: p<0.001). The number of active caspase-3+ cells is not significantly different in the cDKO neocortex at 2 months (p>0.05), but is significantly increased in the cDKO neocortex at 4 months (1.7-fold increase; p<0.01)(n = 4 mice per genotype per age; 10 sections analyzed per mouse). Data are presented as the mean ± s.e.m.

To investigate how neurons die in the absence of presenilins in the adult cerebral cortex, we performed TUNEL analysis to label apoptotic cells (Fig. 2A, B). Indeed, significant increases in TUNEL+ cells were found in the *Psen* cDKO neocortex (2 months: 7.4-fold increase, p<0.00001; 4 months: 15.0-fold increase, p<0.00001; Fig. 2B). Interestingly, no increase was found in TUNEL+ cells in the neocortex of *Psen* cDKO mice at 6 weeks of age (p>0.05; Fig. 2B), despite the fact that presenilin inactivation occurs by postnatal 4 weeks of age, indicating a delay in the onset of apoptotic neuronal death caused by loss of presenilin function.

To confirm further the presence of apoptotic cells, we performed immunostaining using antibodies specific for active (cleaved) forms of caspases 9 and 3, which are excellent markers for apoptosis [19], [20], [21]. At 2 months of age, more active caspase 9-positive cells (7.1±0.3 per 20 µm sagittal section) were found in the *Psen* cDKO neocortex, compared to the control (4.1±0.1 per section; p<0.002; Fig. 2C). Similarly, at 4 months of age, more active caspase 9-positive cells (9.2±1.0 per section) were present in the *Psen* cDKO neocortex, relative to the control (4.2±0.3 per sections; p<0.0003; Fig. 2C). Significant increases of cells that are positive for active caspase 3 were also found in the neocortex of *Psen* cDKO mice (2.7±0.3 per section) at 4 months of age, relative to controls (1.5±0.0 per section, p<0.01; Fig. 2C), while at 2 months the increase of active caspase 3-positive cells in the cDKO neocortex (2.0±0.3) is not significant compared to the control (1.2±0.1, p>0.05; n = 4, 10 sections per brain). These results show that a small percentage of excitatory pyramidal neurons lacking presenilins undergoes apoptosis, as shown by caspase activation and DNA fragmentation, two key features of apoptosis.

### Mild neurodegeneration in the hippocampus of *Psen* cDKO mice

**Figure 3:**
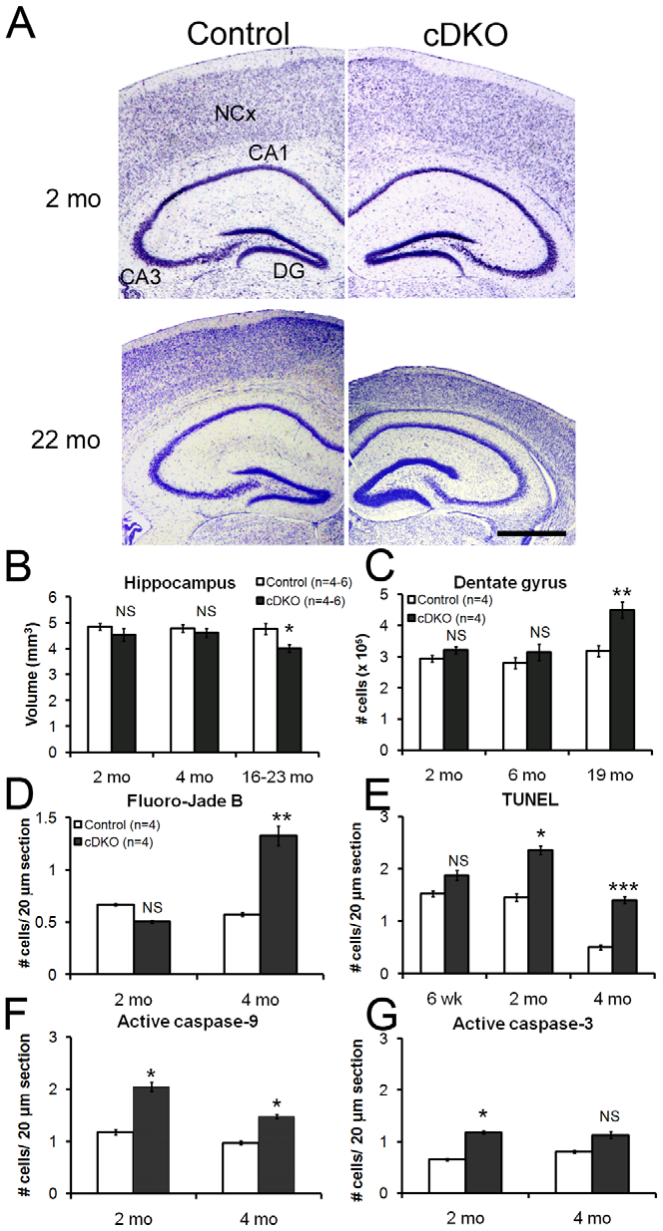
Neurodegeneration and increased adult neurogenesis in the hippocampus of *Psen* cDKO mice. (*A*) Images of Nissl-stained brain sections at 2 and 22 months of age. At 2 months, the neocortex (NCx), hippocampal areas CA1 and CA3, and dentate gyrus (DG) are indistinguishable between *Psen* cDKO and age-matched controls. By 22 months, extensive loss of white and grey matter is evident in the neocortex of *Psen* cDKO mice. In the hippocampus, loss of white matter in cDKO mice coincides with the increase in neurons in the dentate gyrus. Scale bar: 1 mm. (*B*) Stereological quantification of hippocampal volumes. The volume of the *Psen* cDKO hippocampus is normal at 2 months (control: 4.85×10^5^; cDKO: 4.54×10^5^; p>0.05) and 4 months (control: 4.78×10^5^; cDKO: 4.61×10^5^; p>0.05). By 16–23 months, a 15.5% reduction in hippocampal volume is observed (control: 4.76×10^5^; cDKO: 4.02×10^5^; *p<0.05). NS: not significant; n = 4 mice per genotype (2 mo, 4 mo); n = 6 mice per genotype (16–23 mo). (*C*) Stereological quantification of the number of cells within the hippocampal dentate gyrus (DG). Cell number in the DG is comparable between cDKO and control mice at 2 months (control: 2.94×10^5^; cDKO: 3.21×10^5^; p>0.05) and 6 months (control: 2.80×10^5^; cDKO: 3.15×10^5^; p>0.3). In contrast, by 19 months, *Psen* cDKO mice have significantly greater numbers of cells in the DG (control: 3.18×10^5^; cDKO: 4.51×10^5^; **p<0.01)(n = 4 mice per genotype per age). (*D*) Quantification of Fluoro-Jade B+ degenerating neurons. At 2 months, similar numbers of Fluoro-Jade B+ cells are present in control and cDKO mice (p>0.05), in contrast to a 2.3-fold increase in *Psen* cDKO mice by 4 months of age (*p<0.05)(n = 4 mice per genotype per age; 10 sections analyzed per mouse). (*E*) Quantification of apoptotic neurons. Increases in TUNEL+ cells are present in the *Psen* cDKO hippocampus at 2 months (*p<0.05) and 4 months (***p<0.001), whereas the number of TUNEL+ cells is similar in control and cDKO mice at 6 weeks (p> 0.05)(n = 4 mice per genotype per age; 10 sections analyzed per mouse). (*F*) More active caspase-9+ cells are present in the *Psen* cDKO hippocampus at 2 months (p<0.05) and 4 months (p<0.05)(n = 4 mice per genotype per age; 10 sections analyzed per mouse). (*G*) The number of cells positive for active caspase-3+ in the hippocampus is increased in cDKO mice at 2 months (p<0.05), but not significantly increased at 4 months (p>0.05)(n = 4 mice per genotype per age; 10 sections analyzed per mouse). Data are presented as the mean ± s.e.m.

**Figure 4:**
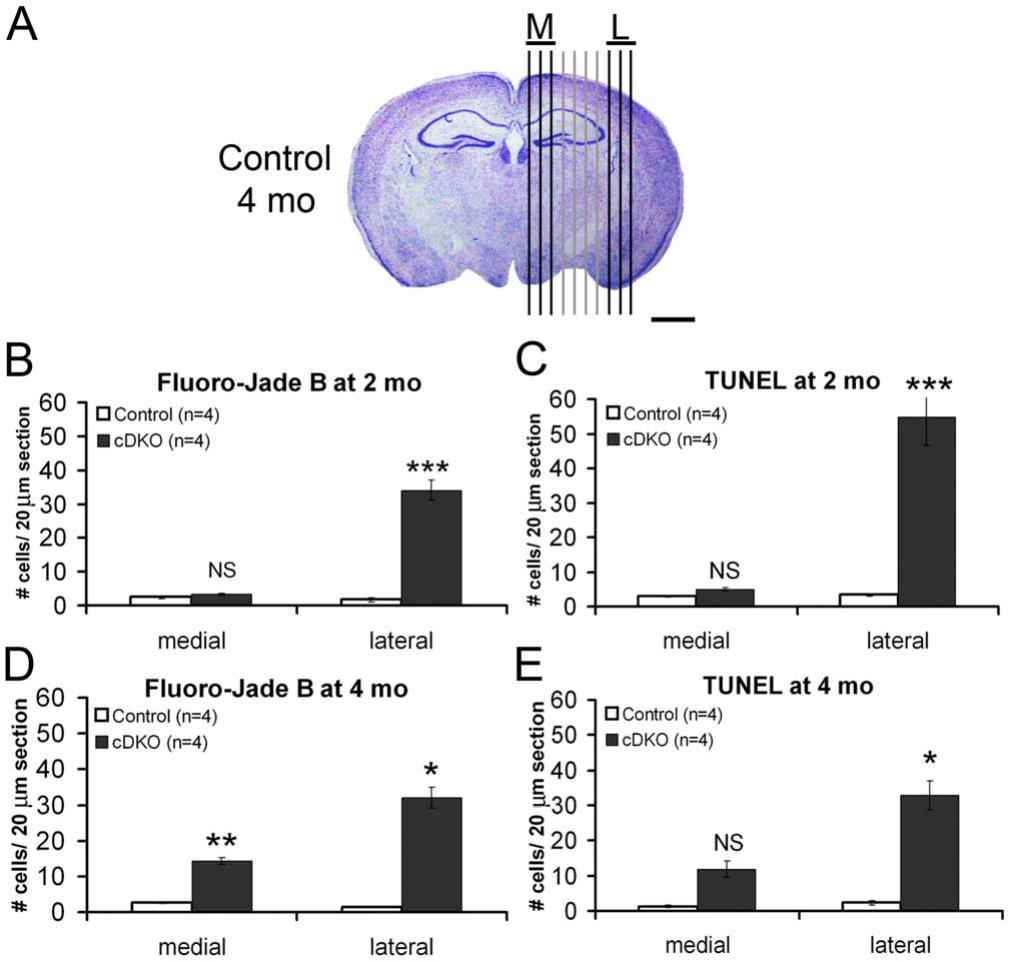
More dramatic increases in apoptosis in the lateral cortex of *Psen* cDKO mice. Black vertical lines superimposed on an image of a Nissl-stained coronal brain section depict the relative positions of sagittal brain sections spaced 300 µm apart that were analyzed for either Fluoro-Jade B+ or TUNEL+ cells. The average (± s.e.m.) of the three medial-most sections (M) was compared to the average of the three lateral-most sections (L). Scale bar: 1 mm. For each timepoint, 4 mice per genotype (10 sections per mouse) were analyzed. (*A, B*) Quantification of numbers of Fluoro-Jade B+ cells in sagittal brain sections from mice at 2 months (*A*) or 4 months (*B*). At 2 months of age, increases in degenerating neurons are more pronounced in the lateral sections (control: 1.7±0.7; cDKO: 34.1±3.1; p<0.01) than in the medial sections (control: 2.5±0.3; cDKO: 3.4±0.3; p>0.05). By 4 months of age, the number of degenerating neurons increases significantly in medial sections of cDKO mice, compared to controls (control: 2.6±0.3; cDKO: 14.3±1.1; p<0.01). Furthermore, the number of degenerating neurons in lateral sections of cDKO mice is much greater than in medial sections (control: 1.4±0.1 vs. *Psen* cDKO: 32.1±3.0; p<0.02). *, p<0.05; **, p<0.01; ***, p<0.001. (*C, D*) Quantification of numbers of TUNEL+ cells in sagittal brain sections from mice age 2 months (*C*) or 4 months (*D*). (*C*) No significant increase was observed in TUNEL+ cells in the *Psen* cDKO medial cortex at 2 months (control: 3.0±0.3 vs. *Psen* cDKO: 4.9±0.5; p = 0.05), in contrast to a large increase in lateral *Psen* cDKO cortex (control: 3.4±0.4 vs. *Psen* cDKO: 54.8±8.0; p<0.05). (*E*) At 4 months, lateral *Psen* cDKO cortex showed a significant increases in TUNEL+ cells (control: 2.3±0.5 vs. *Psen* cDKO: 32.9±4.1; p<0.05), while medial *Psen* cDKO cortex showed only a trend toward increased apoptosis (control: 1.3±0.3 vs. *Psen* cDKO: 11.9±2.3; p = 0.05).

We previously reported that presenilins are essential for neurotransmitter release, NMDAR-mediated functions and long-term potentiation in the hippocampus [10], [22]. We further examined whether loss of presenilins causes age-related neurodegeneration in the hippocampus, similar to the neocortex. Stereological measurement revealed unchanged hippocampal volumes in *Psen* cDKO mice at the ages of 2 months (control: 4.9±0.3 mm^3^, cDKO: 4.5±0.5 mm^3^, p>0.05; Fig. 3A, B) and 4 months (control: 4.8±0.3 mm^3^, cDKO: 4.6±0.3 mm^3^, p>0.05; Fig. 3B). By the age of 16–23 months, the volume of the *Psen* cDKO hippocampus is reduced by 15.5% (control: 4.8±0.5 mm^3^, *Psen* cDKO: 4.0±0.4 mm^3^, p<0.02; Fig. 3A, B), which is much smaller than the ∼53% reduction in the volume of the neocortex.

Contrary to the loss of cortical volume in *Psen* cDKO mice, visual inspection of brain sections suggested an enlargement of the dentate gyrus in the aged *Psen* cDKO hippocampus (Fig. 3A). Stereological quantification confirmed an age-dependent increase in the neuron number in the dentate gyrus (2 mo: control, 2.93×10^5^±0.2×10^5^ vs. cDKO, 3.21×10^5^±0.2×10^5^, p>0.05; 6 mo: control, 2.80×10^5^±0.4×10^5^ vs. cDKO, 3.15×10^5^±0.5×10^5^, p>0.05; 19 mo: control, 3.18×10^5^±0.3×10^5^, vs. *Psen* cDKO, 4.5×10^5^±0.5×10^5^, p<0.01; Fig. 3C). By 19 months of age, *Psen* cDKO mice had 41.5% more neurons in the dentate gyrus of the hippocampus relative to controls, which could be due to an increase in adult neurogenesis in the these brains.

To determine whether *Psen* cDKO hippocampal neurons share a common death mechanism with *Psen* cDKO cortical neurons, we analyzed brain sections with Fluoro-Jade B, TUNEL labeling, and active caspases. In contrast to the sizeable increase in the number of degenerating neurons in the neocortex of *Psen* cDKO mice, a smaller increase is observed in the hippocampus (Fig. 3D–G). Quantitative analysis of Fluoro-Jade B staining revealed no increases in degenerating neurons in the *Psen* cDKO hippocampus at 2 months (p>0.05; Fig. 3D), but a 2.3-fold increase in degenerating neurons at 4 months (p<0.002; Fig. 3D). Similar to the neocortex, no significant increase in TUNEL-positive cells was observed in the hippocampus of *Psen* cDKO mice at 6 weeks (p> 0.05; Fig. 3E), but more TUNEL-positive cells were found in the *Psen* cDKO hippocampus by 2 months (1.6-fold increase, p<0.01; Fig. 3E) and 4 months (2.8-fold increase, p<0.001; Fig. 3E). Consistent with these results, in the *Psen* cDKO hippocampus we observed only mild increases in the number of cells positive for active caspase-9 (Fig. 3F) and active caspase-3 (Fig. 3G). Combined, these data describe a milder apoptotic cell death phenotype in the hippocampus of *Psen* cDKO mice as compared to the neocortex.

### Neurons in the lateral cortex are more susceptible to loss of presenilin function

To determine whether excitatory neurons of the cerebral cortex lacking presenilins exhibit region-specific selective vulnerability, we examined the distribution of degenerating and apoptotic neurons in a series of sagittal brain sections. As shown in Figure 4, the number of Fluoro-Jade B- or TUNEL-positive cells per section in 3 medial (M) or lateral (L) sections was averaged and compared between the genotypic groups. Striking increases were observed in the number of degenerating neurons in the lateral area of the *Psen* cDKO cerebral cortex, comprised of somatosensory and visual cortices, at 2 and 4 months of age (Fig. 4 and Table 2). An average of 20.5-fold increase in Fluoro-Jade B-positive cells was found in the lateral sections of the *Psen* cDKO cortex at 2 months (cDKO: 34.1±3.1 per section; control: 1.7±0.7 per section; Fig. 4B). In the medial area of the *Psen* cDKO cerebral cortex, which consists of motor, anterior cingulate, and retrosplenial cortices, no significant increase in Fluoro-Jade B-positive cells was found at 2 months of age (cDKO: 3.4±0.3 per section; control: 2.5±0.3; p>0.05; Fig. 4B and Table 2). At 4 months of age, *Psen* cDKO mice showed increases (22.6-fold) in Fluoro-Jade B-positive cells in the lateral cortex similar to *Psen* cDKO mice at 2 months, but in contrast to 2 months, the medial cortex of *Psen* cDKO mice at 4 months also showed increased numbers of FJB+ cells (5.5-fold increase; Fig. 4D and Table 2). We further performed the TUNEL assay to identify apoptotic cells and immunohistochemical analysis for active caspases 3 and 9. We found a 16.1-fold increase in TUNEL+ cells in the lateral cortex of *Psen* cDKO mice at 2 months (cDKO: 54.8±8.0 per section; control: 3.4±0.4; p<0.03), but the increase in TUNEL+ cells in the medial cortex was not statistically significant (cDKO: 4.9±0.5; control: 3.0±0.3; 1.6-fold increase; p = 0.05; Fig. 4C). Similarly, at 4 months, a 14.3-fold increase was observed in the lateral cortex of *Psen* cDKO mice (cDKO: 32.9±4.1; control: 2.3±0.5; p<0.05), but the increase in the medial cortex was not statistically significant (cDKO: 11.9±2.3; control: 1.3±0.3; 5.2-fold increase; p = 0.05; Fig. 4E). Similar results were obtained for active caspases (Table 2). These results suggest that compared to medial cortical neurons, lateral cortical neurons are more susceptible to loss of presenilins and are more likely to undergo apoptotic cell death in the absence of presenilins.

**Table 2:** Dying cell distribution along the medial-lateral axis in *Psen* cDKO cortex*.

	FJB at 2 mo			TUNEL at 2 mo			Active casp-9 at 2 mo			Active casp-3 at 2 mo		
Section	Control	cDKO	Fold ↑	Control	cDKO	Fold ↑	Control	cDKO	Fold ↑	Control	cDKO	Fold ↑
1	1.8±2.4	3.0±1.6	**1.7**	3.0±2.4	4.5±1.7	**1.5**	4.5±2.4	6.5±3.4	**1.4**	1.5±1.3	1.3±1.0	**0.8**
2	3.0±2.6	3.3±3.3	**1.1**	3.5±1.3	4.3±1.3	**1.2**	3.0±1.4	4.8±1.5	**1.6**	0.8±1.0	1.3±0.5	**1.7**
3	2.8±2.8	4.0±2.9	**1.5**	2.5±1.0	6.0±2.3	**2.4**	2.8±1.5	4.8±1.7	**1.7**	0.5±1.0	0.8±1.5	**1.5**
4	2.3±1.7	5.8±2.6	**2.6**	2.0±1.4	10.0±4.1	**5.0**	6.0±2.2	5.3±1.5	**0.9**	1.5±1.9	1.3±1.3	**0.8**
5	1.3±1.5	10.8±5.9	**8.6**	5.3±2.2	16.5±3.3	**3.1**	5.8±3.3	3.5±1.3	**0.6**	1.0±1.4	1.0±0.8	**1.0**
6	2.8±2.2	12.0±8.0	**4.4**	5.0±3.5	27.8±13.9	**5.6**	3.8±2.9	6.3±1.7	**1.7**	2.0±0.8	1.8±1.7	**0.9**
7	2.3±3.3	18.0±12.2	**8.0**	4.5±2.4	29.0±7.1	**6.4**	4.3±2.5	4.8±2.9	**1.1**	0.8±0.5	1.3±2.5	**1.7**
8	3.3±1.0	27.3±24.1	**8.4**	4.0±1.6	36.5±22.5	**9.1**	4.3±1.5	7.0±6.0	**1.7**	1.5±0.6	4.5±4.5	**3.0**
9	1.0±1.2	36.0±28.4	**36.0**	3.8±1.5	61.3±28.4	**16.3**	2.5±2.1	10.5±7.1	**4.2**	0.8±1.0	5.5±5.8	**7.3**
10	0.8±0.5	39.0±31.2	**52.0**	2.5±1.7	66.5±29.3	**26.6**	3.5±2.5	18.5±7.9	**5.3**	1.8±1.0	1.8±2.4	**1.0**

	FJB at 4 mo			TUNEL at 4 mo			Active casp-9 at 4 mo			Active casp-3 at 4 mo		
Section	Control	cDKO	Fold ↑	Control	cDKO	Fold ↑	Control	cDKO	Fold ↑	Control	cDKO	Fold ↑
1	2.3±1.3	16.8±7.0	**7.4**	2.0±2.2	8.0±3.9	**4.0**	6.0±3.4	1.0±1.4	**0.17**	0.8±1.0	2.5±1.3	**3.3**
2	2.3±0.5	13.0±5.4	**5.8**	1.0±1.2	10.8±9.7	**10.8**	3.0±2.6	4.5±3.9	**1.5**	1.5±1.3	0.8±1.0	**0.5**
3	3.3±2.1	13.3±1.7	**4.1**	0.8±0.5	17.0±5.8	**22.7**	4.8±3.6	5.8±5.3	**1.2**	2.0±1.2	2.0±3.4	**1.0**
4	4.3±1.7	14.3±2.8	**3.4**	1.0±0.8	16.5±8.9	**16.5**	4.8±2.9	6.8±8.5	**1.4**	1.5±1.0	2.8±2.8	**1.8**
5	3.8±1.7	22.5±5.2	**6.0**	1.8±1.3	21.5±12.3	**12.3**	4.0±2.5	10.3±6.8	**2.6**	1.8±1.7	3.0±1.8	**1.7**
6	2.0±0.0	28.3±2.6	**14.1**	2.0±0.8	40.3±14.9	**20.1**	4.5±2.4	13.0±7.4	**2.9**	0.8±1.0	3.0±3.2	**4.0**
7	3.5±2.4	26.0±5.9	**7.4**	0.8±0.5	30.0±5.5	**40.0**	2.8±1.7	11.8±6.8	**4.3**	1.5±0.6	2.5±1.3	**1.7**
8	1.3±0.5	38.5±5.5	**30.8**	3.5±2.1	33.0±17.6	**9.4**	4.5±1.3	16.3±13.0	**3.6**	1.5±1.0	3.3±2.6	**2.2**
9	1.8±1.7	26.8±10.4	**15.3**	1.8±1.5	41.0±18.8	**23.4**	5.5±1.3	10.3±5.9	**1.9**	1.5±0.6	4.0±2.9	**2.7**
10	1.3±1.9	31.0±7.0	**24.8**	1.5±0.6	24.8±10.9	**16.5**	2.3±2.5	12.5±5.8	**5.6**	2.5±2.1	2.8±1.7	**1.1**

*FJB*, Fluoro-Jade B; *TUNEL*, terminal deoxynucleotidyl transferase dUTP nick end labeling; *casp*, caspase; ↑, increase.

*Values are expressed as the average number of positive cells per 20 µm-thick sagittal section; standard deviations are indicated (n = 4 mice per genotype). Sections are listed from medial (#1) to lateral (#10). Adjacent sections are separated by a distance of approx. 300 µm (total distance = 2.7 mm).

### Mitochondrial defects in the *Psen* cDKO cortex

**Figure 5:**
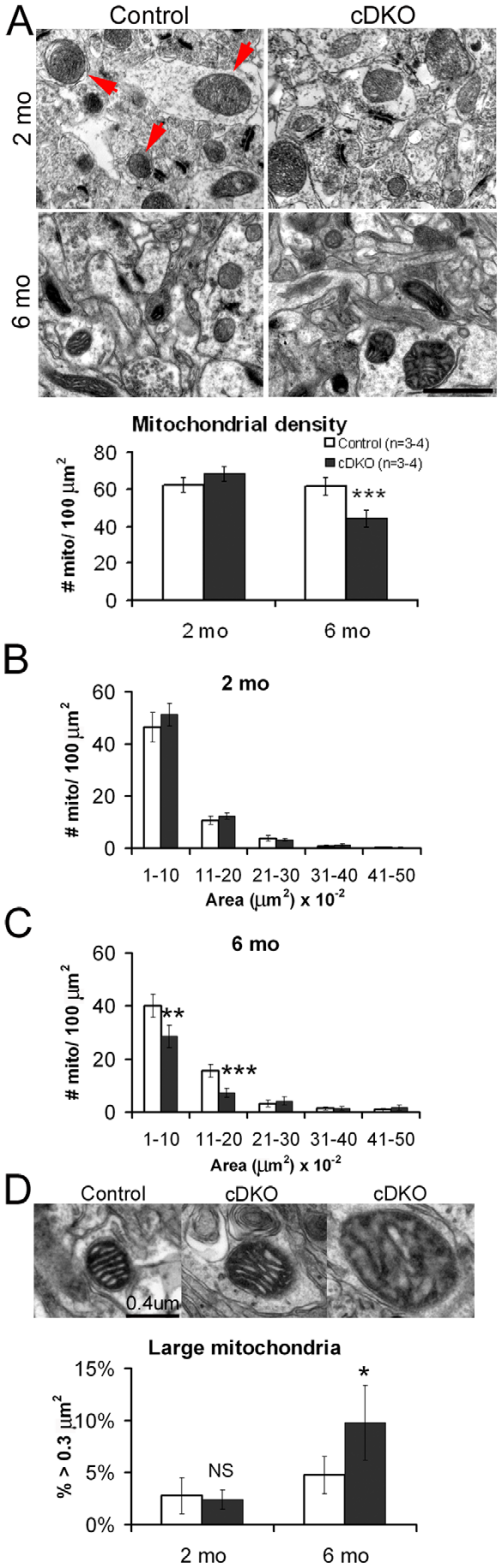
Mitochondrial defects in the cerebral cortex of *Psen* cDKO mice. (*A*) (*Top*) Electron micrographs of mitochondria from the neocortex at 2 months and 6 months of age. Red arrows point to mitochondria typical of those quantified in (*A–C*). Scale bar, 1 µm. (*Bottom*) Quantification of mitochondria per 100 µm^2^ in neocortex. The density of *Psen* cDKO mitochondria was comparable to controls at 2 months (control: 62.5±4.1 vs. *Psen* cDKO: 68.4±4.1; p>0.05) but reduced by 6 months (control: 61.7±4.8 vs. *Psen* cDKO: 44.2±4.6; p<0.001), revealing age-dependent loss of mitochondria in *Psen* cDKO (n = 4 mice per genotype (2 mo) and 3 mice per genotype (6 mo)). (*B,C*) Analysis of size distribution of cortical mitochondria determined that *Psen* cDKO has no change in mitochondrial morphology at 2 months (*B*), but a sizeable reduction in small mitochondria by 6 months of age (*C*) (0.01–0.1 µm^2^ bin: control, 40.0±4.4 vs. *Psen* cDKO, 28.6±4.3 (p<0.001); 0.11–0.2 µm^2^ bin: control, 15.5±2.3 vs. *Psen* cDKO, 7.3±1.6 (p<0.00001)). (*D*) (*Top*) High-magnification electron micrographs of cortical mitochondria from mice at 6 months of age. Both control (*left*) and *Psen* cDKO samples (*middle*) have normal mitochondria with clearly visible cristae and outer membrane structures; however, *Psen* cDKO mice also have a slight increase in the number of swollen mitochondria (*right*). Scalebar, 0.4 µm (all 3 images). (*Bottom*) Quantification of large (area >0.3 µm^2^) mitochondria from mice age 2 months and 6 months. The percentage of large *Psen* cDKO cortical mitochondria relative to the total number was normal at 2 months of age (control: 0.028±0.017 vs. *Psen* cDKO: 0.024±0.009; p>0.05), but at 6 months of age, the percentage of large *Psen* cDKO mitochondria is twice the number observed in controls (control: 0.048±0.018 vs. *Psen* cDKO: 0.098±0.035; p<0.05), implicating a dysregulation of mitochondrial morphology in the absence of PS function. (n = 4 at 2 months and 3 at 6 months). All data are presented as the mean ± s.e.m.

Since Presenilins are present in the mitochondrial/lysosomal fraction of the brain [22], and normal mitochondrial function is essential to neuronal viability, we examined the consequence of presenilin inactivation in mitochondria. We quantified the density of mitochondria in the neocortex at 2 and 6 months of age using electron microscope (EM) images (Fig. 5A). Quantitative analysis revealed that numbers of mitochondria per 100 µm^2^ in the *Psen* cDKO neocortex are normal at 2 months (control: 62.5±4.1; cDKO: 68.4±4.1; p>0.05) but reduced at 6 months (control: 61.7±4.8; cDKO: 44.2±4.6; p<0.001; Fig. 5A). More detailed analysis of mitochondrial size, or quantification of the area of each mitochondrion and assignment into arbitrary bins of 0.1 µm^2^ (Fig. 5B, C), revealed normal size distribution of *Psen* cDKO mitochondria at 2 months (Fig. 5B), but decreased numbers of smaller mitochondria (<0.2 µm^2^) in the neocortex of *Psen* cDKO mice at 6 months of age (Fig. 5C). In addition to the decrease in smaller mitochondria in the *Psen* cDKO neocortex, we observed an increase in larger mitochondria (Fig. 5D). Quantitative analysis of mitochondria revealed a higher percentage of larger mitochondria (>0.3 µm^2^) in the *Psen* cDKO neocortex (9.8%±3.6), compared to controls (4.7%±1.8; p<0.05; Fig. 5D) at 6 months of age. Combined, the reduced mitochondrial density and altered size distribution in the aging *Psen* cDKO cortex may contribute to neuronal degeneration observed in these mice.

## Discussion

### Loss of presenilins causes a delayed onset of apoptotic cell death in the cerebral cortex

In *Psen* cDKO mice, *Presenilin* inactivation is dependent on expression of Cre recombinase under the control of the *αCaMKII* promoter, Cre-mediated recombination of the floxed *Psen1*, and turnover of *Psen1* mRNAs and proteins. Our *in situ* hybridization and western analyses have shown that expression of *Cre* mRNAs and loss of *Psen1* mRNAs and proteins begins at 3 weeks of age in the cerebral cortex ([14]; also MWS and JS, unpublished data).

A striking feature of *Psen* cDKO mice is the delayed onset of apoptosis and degeneration relative to the timing of presenilin inactivation. As opposed to mouse models of hypoxia-ischemia, or hypoglycemia, or exposure to excitotoxins (e.g. glutamate) [23], [24], [25] or other neurotoxic compounds such as ethanol or NMDA antagonists [26], [27], [28], [29], where the brain has a rapid, dramatic increase in dying cells, we failed to detect any increases in TUNEL+ cells in the cerebral cortex of *Psen* cDKO mice at 6 weeks of age (Figure 2), despite the presence of presynaptic and postsynaptic defects at this age (D. Zhang and JS, unpublished results). By 2 months of age, 5 weeks after the onset of presenilin inactivation, significant increases in apoptotic and degenerating neurons were found in the cerebral cortex of *Psen* cDKO mice (Figures 1 and 2). However, no significant reduction of cortical volume, which reflects the loss of neuronal processes as well as neuronal numbers, and cortical neuron number was found at 2 months of age, because of the low absolute number of cells that are undergoing apoptosis at this age (see below).

### Apoptotic cell death occurs in a very small percentage of cortical neurons lacking presenilins

Cortical neurons lacking presenilins degenerate at a slow steady rate of approximately 0.1% of total neurons, measured by TUNEL+ and Fluoro-Jade B+, beginning at 2 months of age (Table 1). This rate corresponds to the percentage of cells undergoing apoptotic death at the time of the detection, since the time course of apoptosis is very short and dying cells are usually removed within 12–24 hours [30], [31]. Interestingly, this low rate of cortical neurons undergoing apoptosis we have identified in *Psen* cDKO mice is similar to the observed rate of apoptotic cell death (0.02–0.09%) in AD brains [30], [32], [33], [34].

### Progressive loss of neurons and neuronal processes in the cerebral cortex of *Psen* cDKO mice

Although only a very small percentage of cortical neurons lacking presenilins would undergo apoptotic cell death, the number of cells lost accumulates over time, leading to significant loss of cortical neurons in the aging cerebral cortex. For example, at 2 months of age, dramatic increases in apoptotic cells were detected in *Psen* cDKO mice, compared to controls, but no significant loss of cortical volume and neurons was found (Figure 1). However, by 4 months of age, significant loss of cortical volume (∼22%) and neurons (∼9%) was present (Figure 1). This degeneration process continues, leading to loss of ∼18% and 24% of cortical neurons at 6 and 9 months of age, respectively (Table 1). This slowly progressive nature of cortical neuronal degeneration observed in *Psen* cDKO mice further highlights the relevance of this model to the study of similarly slow, progressive neuronal degeneration in AD.

The delay of onset in cell death and the low rate of apoptotic cell death among cortical neurons lacking presenilins suggest that loss of presenilins may sensitize cells to die rather than immediately triggering apoptosis. While the identity of the crucial death trigger is unclear, the apoptotic cell death could be induced by a disruption of cellular homeostasis caused by loss of presenilins, which could in turn affect the survival of cortical neurons to varying degrees.

### Region specificity of vulnerability of cortical neurons to loss of presenilins

A common characteristic of Alzheimer's disease and other neurodegenerative diseases such as Parkinson's and Huntington's is the region specificity of neuronal degeneration in the brain. In the case of AD patients, neuronal loss is most prominent in the hippocampus, the temporal cortex (lateral), and the frontal cortex. Recent functional brain imaging (MRI) has found that the earliest signs of altered brain activity in families with AD are decreased activity in the cingulate cortex and decreased connectivity between the hippocampus and cingulate cortex [35], [36]. Interestingly, the lateral cortex of *Psen* cDKO mice, which includes visual and cingulate cortices, indeed shows the earliest and most striking neuronal death (Figure 4). However, while loss of presenilins causes hippocampal dependent learning and memory deficits as well as impaired synaptic functions in hippocampal neurons [10], [14], [22], the number of hippocampal neurons was not significantly decreased (CA Saura, MWS & JS, unpublished data). Increases in apoptotic cell death were detected in the hippocampus of *Psen* cDKO mice at 2 and 4 months of age (Figure 3), but the increase was less dramatic than that in the neocortex of *Psen* cDKO mice (Figures 1 and 2). It is not entirely clear why the total number of pyramidal neurons in the hippocampus of *Psen* cDKO mice is not significant reduced, though loss of dendritic complexities [10] and loss of hippocampal volumes (Figure 3) were seen in aged *Psen* cDKO mice. One likely possibility is increased adult neurogenesis occurring in the hippocampus, which would compensate for the age-related loss of hippocampal neurons in the absence of presenilins. The fact that we indeed found a 41% increase in the number of neurons in the dentate gyrus of *Psen* cDKO mice at 19 months, the site of adult neurogenesis, lends further support to this interpretation. The exact mechanism underlying increased neurogenesis in the aging dentate gyrus of *Psen* cDKO mice is unknown. Since presenilin inactivation is targeted specifically to postmitotic excitatory neurons, rather than neural stem cells, this increase in neurogenesis is likely to be secondary to functional defects and neurodegenerative processes occurring in the hippocampus of *Psen* cDKO mice. Furthermore, loss of presenilin in neural progenitor cells during development results in decreases, rather than increases, in the number of neurons generated, due to premature depletion of neural progenitor pool in the absence of presenilins [5], [6], further arguing against a cell autonomous direct effect.

In summary, we found that loss of presenilins in mature excitatory neurons in the mouse cerebral cortex results in increases in apopototic cell death among a very small percentage of cortical neurons at any given time. Over time, this low rate of cell death leads to significant loss of cortical neurons in an age dependent and region specific manner, reminiscent of the slow and progressive neurodegeneration process in AD. Since mutations in presenilins likely cause AD at least in part *via* a partial loss of function mechanism [37], this mouse model provides a unique experimental tool to study further the molecular mechanisms underlying neurodegeneration caused by loss of presenilin function and in FAD.
